# Apligraf as an Alternative to Skin Grafting in the Pediatric Population

**DOI:** 10.7759/cureus.16226

**Published:** 2021-07-07

**Authors:** Morgan Eudy, Christi L Eudy, Samuel Roy

**Affiliations:** 1 Surgery, Campbell University School of Osteopathic Medicine, Salisbury, USA; 2 Plastic and Reconstructive Surgery, Piedmont Plastic and Oral Surgery, Salisbury, USA

**Keywords:** bioengineered living cell constructs, apligraf, split thickness skin graft, skin graft, full thickness wounds, full thickness skin graft, pediatric plastic surgery

## Abstract

Split-thickness skin grafting and healing by secondary intention are the most common options for the treatment of full-thickness skin injuries. This case explores Apligraf (Organogenesis Inc., Canton, Massachusetts) as an alternative treatment for full-thickness skin injuries in the pediatric population. Apligraf, a bioengineered living cell construct, is an advanced wound care modality that is commonly used to treat chronic, nonhealing venous leg ulcers and diabetic foot ulcers. This case demonstrates Apligraf as a viable, if not superior, treatment option for full-thickness skin injury.

## Introduction

Bioengineered living cell constructs (BLCC), such as Apligraf (Organogenesis Inc., Canton, Massachusetts), are approved by the Food and Drug Administration (FDA) for the treatment of chronic, nonhealing venous leg ulcers (VLU) and diabetic foot ulcers (DFU) [1]. Apligraf is a living, bi-layered skin substitute formed by human neonatal foreskin-derived keratinocytes and fibroblasts in a bovine type 1 collagen lattice [1]. Studies looking into the mechanism of action of BLCC have demonstrated that they are able to phenotypically convert chronic nonhealing wounds to a more acute inflammatory environment that can promote healing [1]. This is achieved by inducing genes that are responsible for acute inflammation healing, stimulating wound edge keratinocytes, and attenuating Wnt/β-catenin [1].

## Case presentation

**Figure 1:**
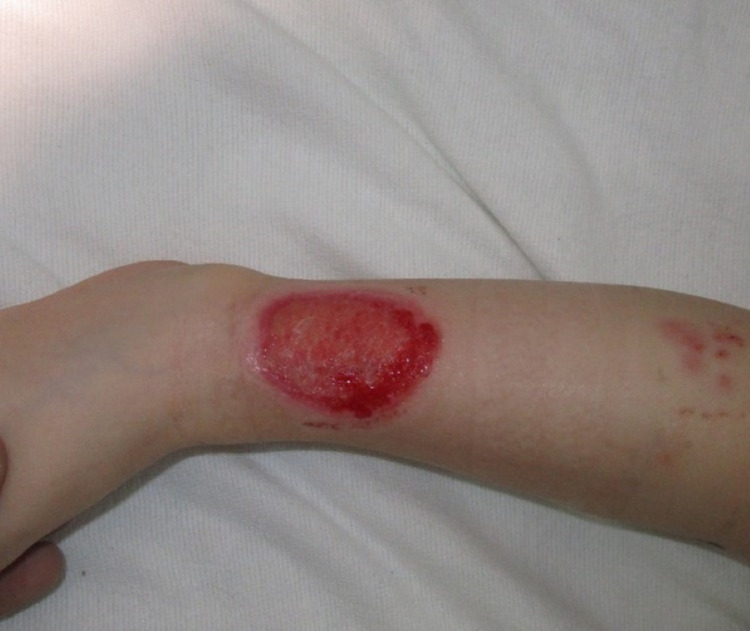
Initial consultation at the plastic surgeon’s office

Our patient, a three-year-old female, presented after a fall on a treadmill, resulting in a 5 cm x 4 cm full-thickness skin injury on the right, distal 1/3rd of the leg (Figure 1). She was initially treated with silver sulfadiazine, and a split-thickness skin graft (STSG) was planned for definitive treatment. The patient’s family consulted a plastic surgeon searching for additional treatment options, with specific concerns about cosmetics and creating a donor site scar.

At this point, the options for treatment included STSG, healing by secondary intention, and advanced modalities such as Apligraf. After discussing the options with the patient’s family, they elected Apligraf.

**Figure 2:**
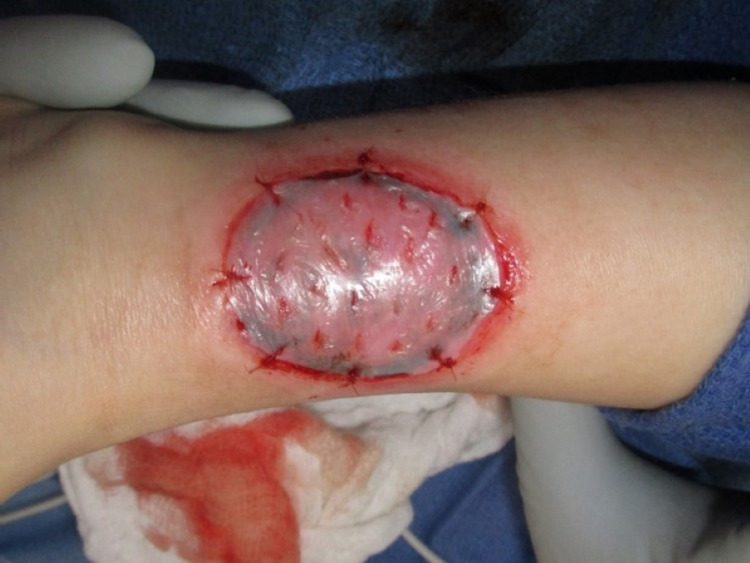
Immediately after the procedure, with the initial Apligraf in place

To place Apligraf, the patient was taken to the operating room and under general anesthesia, the wound was first debrided sharply and irrigated using pulsa-vac and then the wound bed was cauterized for hemostasis. Apligraf was fenestrated make the membrane more permeable, applied to the wound, and secured using multiple resorbable sutures as shown in Figure 2. A wound vacuum-assisted closure (VAC) dressing was placed for five days.

**Figure 3:**
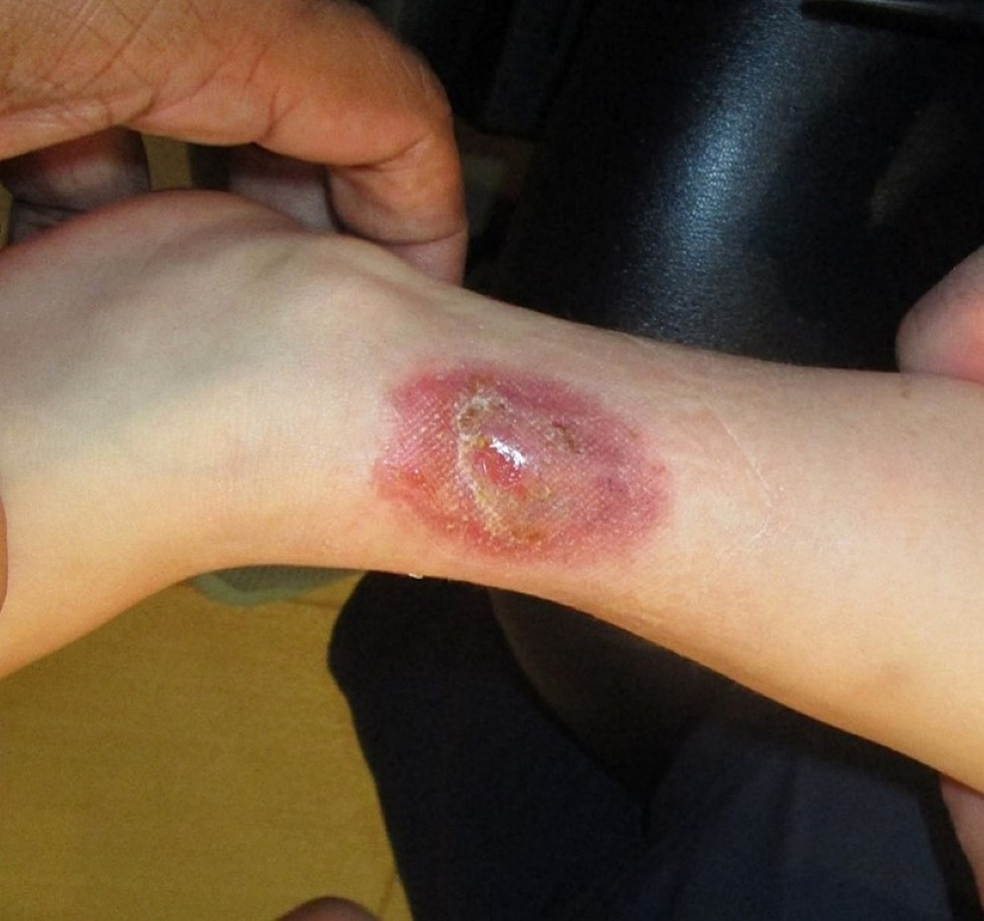
Postoperative day 39, after the application of three Apligrafs

**Figure 4:**
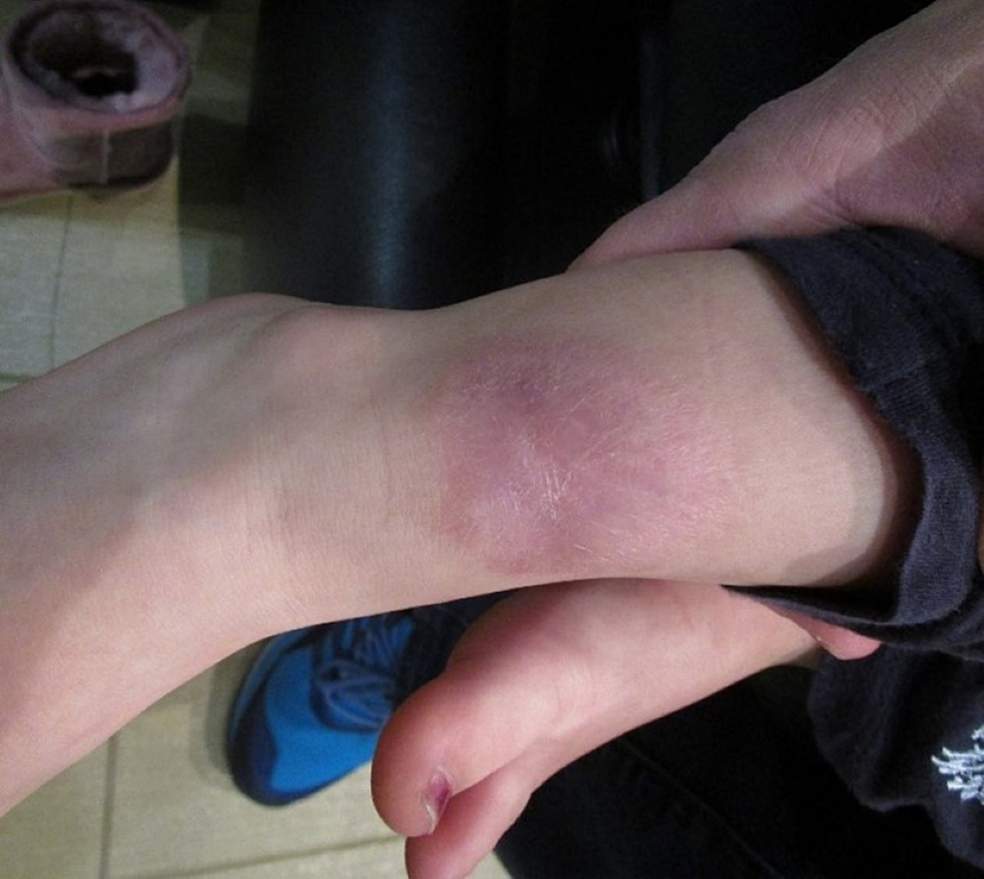
Eleven months postoperation

Apligraf was reapplied in the office on postoperative days 12 and 24 and held in place with Steri-Strips, Adaptic (vaseline gauze), 4x4 gauze, Kerlix, and Coban. Figure 3 shows postoperative day 39, after the application of the three total Apligrafs. Figure 4 shows the complete resolution of the full thickness injury 11 months after the initial placement of the Apligraf.

## Discussion

This case demonstrates Apligraf as a valid alternative to STSG in full thickness skin injuries. While a STSG is typically the treatment of choice for wounds such as in this case, there are a few draw backs. Autografts require the creation of a donor site, adding an additional scar and recovery time. The other treatment option that was proposed to the family was healing by secondary intention. The downside to this approach was the possible development of a severe wound contracture and the functional implications this could have to the patient later in life. The cosmetic appearance associated with the creation of a donor site or healing via secondary intention were a major concern for the patient’s family and the reason they opted for an alternative modality.

While Apligraf is FDA approved for VLU and DFU, it has been used in several other clinical scenarios. One study used Apligraf in surgical wound defects after skin cancer removals as an alternative to skin grafting or healing by secondary intention [2]. While the participation of the study was low (17 adults) and there was no control group, the authors of the study concluded that they were pleased with the cosmetic results of using Apligraf [2]. The authors felt there was improved healing when using the Apligraf versus healing by secondary intention [2].

In the pediatric population, Apligraf has been used in the treatment of epidermolysis bullosa. One study of 9 pediatric patients found the use of Apligraf resulted in rapid healing, easier wound care, improved ambulation and dexterity versus skin grafting [3].

Despite the positive results surrounding the use of Apligraf, there are very limited studies and cases of its use in the pediatric population. Additional studies need to be conducted to fully compare the use of Apligraf versus STSG and healing by secondary intention in pediatric patients. An additional consideration for providers and patients considering Apligraf is cost. According to the 2021 Medicare rates from Organogenesis website, Apligraf costs anywhere from approximately $870-$3,500 depending on multiple factors. Apligraf is not currently covered by insurance outside of its FDA approved indications of VLU and DFU.

## Conclusions

This case brings to light the pros and cons of the standard treatment of full thickness skin injuries, with an emphasis on cosmetic concerns. Apligraf is a viable option for treatment of burns and produces pleasing cosmetic results in the pediatric population.
